# Delayed-onset diffuse lamellar keratitis due to retained SMILE lenticule: A case report and literature review

**DOI:** 10.1097/MD.0000000000042095

**Published:** 2025-04-18

**Authors:** Guangbi Fu, Jiaxing Pan, Hai Yu, Wei Yang, Zhengzheng Wu

**Affiliations:** a Department of Ophthalmology, The Affiliated Hospital of Southwest Medical University, Luzhou, Sichuan, China; b Department of Ophthalmology, Deyang People’s Hospital, Deyang, Sichuan, China; c Department of Ophthalmology, Sichuan Academy of Medical Sciences and Sichuan Provincial People’s Hospital, School of Medicine, University of Electronic Science and Technology of China, Chengdu, China.

**Keywords:** corneal surgery complications, corneal transparency, delayed-onset diffuse lamellar keratitis, small incision lenticule extraction, visual recovery

## Abstract

**Rationale:**

Delayed-onset diffuse lamellar keratitis is a rare but serious complication that can occur after small incision lenticule extraction surgery. Retained lenticules may act as foreign bodies, potentially triggering inflammatory reactions in the cornea.

**Patient concerns:**

A 30-year-old Chinese woman was referred for ophthalmological assessment due to declining vision in both eyes for more than 10 years. She had been wearing glasses for 4 years and contact lenses for 2 years.

**Diagnoses:**

The patient was diagnosed with myopia.

**Interventions:**

The patient underwent uneventful small incision lenticule extraction surgery in the right eye but did not get lenticule extracted in the left eye. Then she developed symptoms of keratitis in the left eye on postoperative day 5, characterized by diffuse gray-white stromal opacities. Immediate treatment with anti-inflammatory medications and irrigation effectively controlled the inflammation. Subsequently, a second lenticule extraction procedure was performed.

**Outcomes:**

Corneal transparency and visual clarity restored in the left eye. The visual acuity of both eyes improved to 1.2 at 3 months after surgery.

**Lessons:**

This case report emphasizes the link between delayed-onset diffuse lamellar keratitis and retained lenticules, which may act as foreign bodies triggering inflammatory reactions in the cornea. Clinicians should diagnose and treat this keratitis promptly to support successful visual recovery and patient satisfaction. Further research should explore the pathogenesis and risk factors to improve management strategies.

## 1. Introduction

Diffuse lamellar keratitis (DLK) is a common complication following refractive surgeries like LASIK and SMILE, characterized by inflammation in the corneal stromal layer, leading to opacity and decreased vision.^[1]^ While typically seen shortly after surgery, delayed-onset DLK, appearing days to weeks postoperatively, is also reported.^[2]^

Small incision lenticule extraction (SMILE) surgery, an advanced refractive procedure, involves extracting a corneal lenticule through a small incision to correct vision.^[3]^ Compared to LASIK, SMILE offers advantages such as smaller incisions, quicker recovery, and reduced dry eye symptoms.^[4,5]^ However, it can still lead to DLK, likely due to factors like microbial contamination, mechanical trauma, or immune responses.

Recent studies suggest retained lenticules in the cornea post-SMILE may trigger delayed-onset DLK by causing mechanical irritation or interacting with corneal tissue, inducing inflammation.^[6]^ Understanding these mechanisms is crucial for improving DLK prevention and management strategies in SMILE surgery, thereby enhancing patient outcomes. This study aims to explore the relationship between delayed-onset DLK and retained lenticules, providing insights for clinical practice.

## 2. Case description

Ms. Cai, a 30-year-old patient, presented to our clinic with declining vision in both eyes and a diagnosis of myopia exceeding 10 years. She had been wearing glasses for 4 years and contact lenses for 2 years. The primary complaints included a visual acuity of only 0.06 in both eyes without correction and abnormal refractive measurements upon dilation. Corneal curvature and thickness measurements also indicated significant changes. Comprehensive eye examinations, including visual acuity, refraction, and corneal measurements, were conducted, with detailed results provided in Table 1. Based on these findings, refractive surgery was recommended, and a preoperative preparation and postoperative monitoring plan was established. All procedures were approved by the ethics committee, and the patient provided informed consent.

**Table 1:** Preoperative eye examination results.

Parameter	Right eye	Left eye
Naked visual acuity	0.06	0.06
Cycloplegic refraction	−3.75 = 1.0	−3.75/−0.25 × 15 = 1.0
Refraction	−3.75 = 1.0	−3.75/−0.25 × 15 = 1.0
Corneal curvature	45.5 × 167	45.1 × 21
	46.0 × 77	46.0 × 111
Corneal thickness (um)	539	533
Corneal diameter (mm)	11.2	11.3
Pupil size (mm)	5.3	6.1
Intraocular pressure (mm Hg)	15	16
Kappa angle	*R* = 0.17@91	*R* = 0.24@131

**Figure 1.**
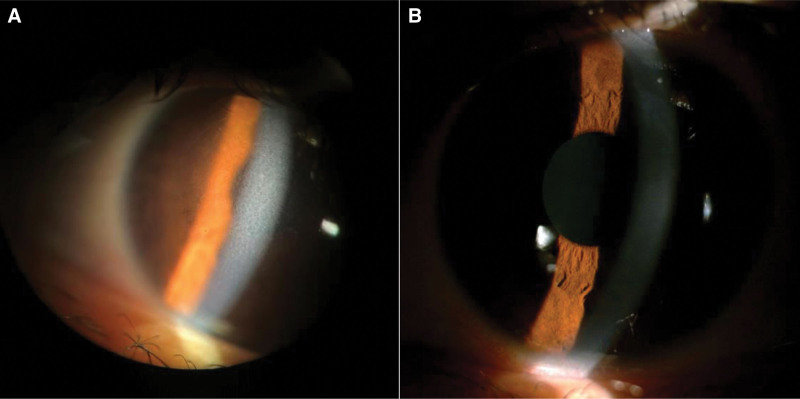
Slit-lamp microscopy images of the cornea. (a) Preoperative corneal image: (b) postoperative corneal image.

**Figure 2.**
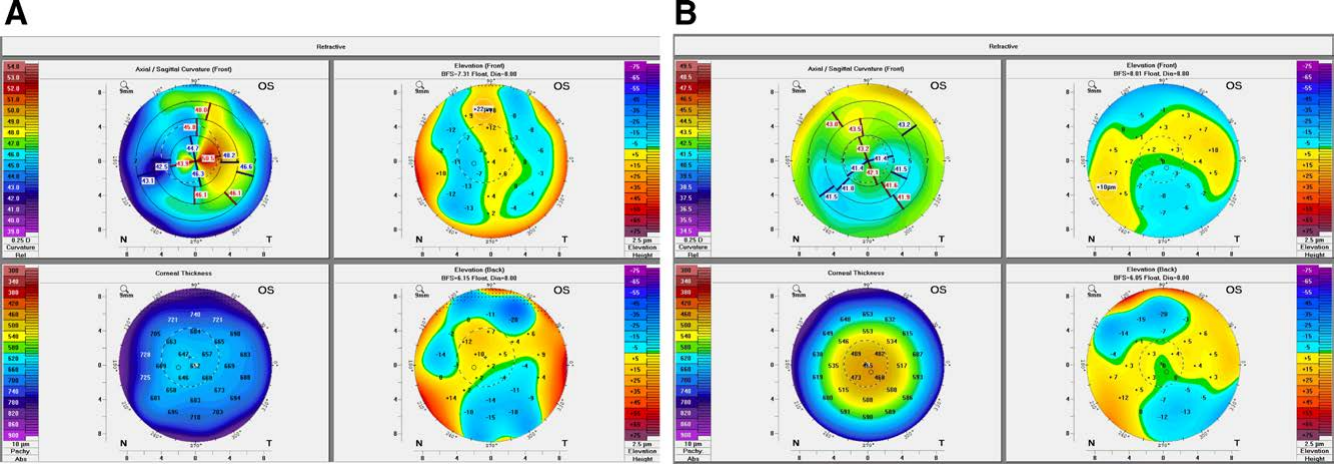
Postoperative corneal topography and parameters on days 5 and 80. (a) Day 5 postoperative measurements; (b) day 8 postoperative measurements.

**Figure 3.**
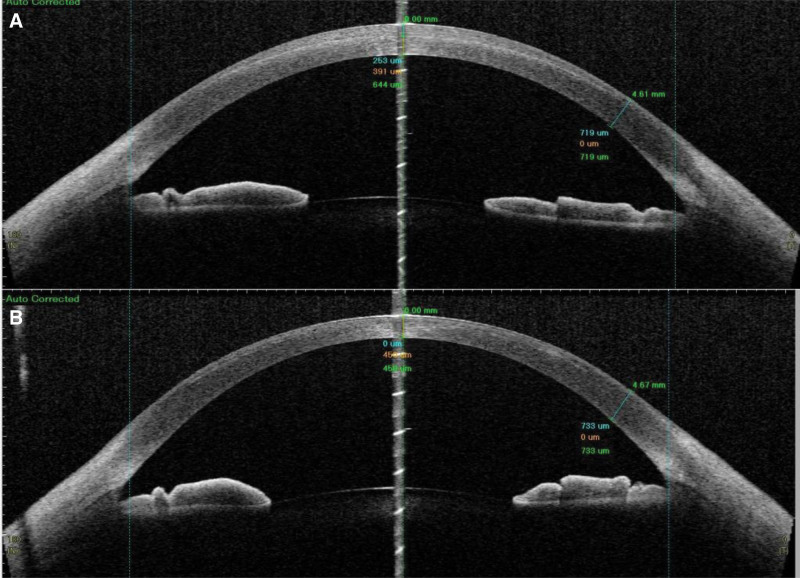
Corneal thickness and shape evolution post-surgery. (a) Corneal thickness 5 days after the first surgery; (b) corneal thickness 80 d after the second surgery.

The visual acuity of the right eye improved from 1.0 on the first day to 1.2 at 3 months, while the left eye’s visual acuity increased from 0.02 on the first day to 1.2 at 3 months. Throughout the treatment period, the management plan was adjusted over time, incorporating various medications and procedures to promote corneal healing and improve visual acuity. The treatment process is detailed in Table 2. During the subsequent 3 months of treatment, the corneal transparency of the right eye remained clear throughout the follow-up period, while the left eye initially exhibited significant opacity, which improved with treatment. Significant edema was observed in the left eye initially, but it gradually resolved with adjustments to the treatment plan and a second lenticule extraction procedure (Fig. 1). The corneal curvature underwent significant changes throughout the postoperative period, with initial alterations following surgery and further adjustments noted after the second lenticule extraction. By the 3-month follow-up, the corneal curvature had stabilized, showing improvements and achieving values closer to the desired outcomes (Fig. 2). During the postoperative period, the corneal curvature underwent significant changes. Initially, there were notable alterations shortly after the surgery, and further adjustments were observed following the second lenticule extraction. By the 3-month follow-up, the corneal curvature had stabilized, showing improvement and nearing the expected outcome. The corneal thickness is measured at different points in the image taken 5 days after the first surgery, showing values such as 253 µm, 391 µm, and 644 µm. In the image taken 80 days after the second surgery, the corneal thickness is recorded at 733 µm. This indicates that over time, the cornea has undergone some degree of recovery, with an increase in thickness. Additionally, the corneal shape has changed between the 2 postoperative periods, particularly in the central and peripheral regions. After 80 days, the corneal shape appears smoother and more symmetrical. The increase in corneal thickness from 644 µm at 5 days to 733 µm at 80 days suggests that the cornea may have experienced some degree of rebound or healing, leading to this thickening (Fig. 3).

**Table 2:** Changes in patient condition and treatment process.

Date	Vision (right/left eye)	Corneal status	Intraocular pressure	Refraction results	Treatment plan	Notes
Day 1 postoperative	1.0/0.02	Partial return of corneal transparency with edema	Normal	Mild myopia in the right eye; significant myopia and astigmatism in the left eye	Tobradex eye drops, Cozzar, Besivance, sodium hyaluronate, Alphagan, bandage lens	Patient reported only slight tearing
Day 2 postoperative	1.0/0.02	Significant corneal edema in the left eye	Normal	Mild myopia in the right eye; significant myopia and astigmatism in the left eye	Increased Tobradex eye drops to 8 times per day	
Day 5 postoperative	1.0/0.6	Clear in the right eye; diffuse grayish-white opacity in the left eye	Normal	Mild myopia in the right eye; significant myopia and astigmatism in the left eye	Fluorometholone, Cozzar, Besivance, sodium hyaluronate, Alphagan, removal of bandage lens, intraocular irrigation, Tobradex eye drops every 2 h	Slight refractive changes in the right eye; considerable changes in the left eye
Day 8 postoperative	1.0/0.6	Clear cornea	Normal	Mild myopia in the right eye; significant myopia and astigmatism in the left eye	Second lenticule extraction	
Day 9 postoperative (day 1 after second lenticule extraction)	1.0/0.8	Clear cornea with no significant edema	Normal	Mild myopia in the right eye; significant myopia and astigmatism in the left eye	Fluorometholone, Cozzar, Besivance, sodium hyaluronate, Alphagan, Tobradex eye drops, levofloxacin eye drops	Patient reported no discomfort
Day 23 postoperative (day 13 after second lenticule extraction)	1.0/1.0	Clear cornea	Normal	Mild myopia and slight astigmatism in both eyes	Fluorometholone (twice daily), Cozzar, Besivance, sodium hyaluronate, Alphagan	
3 mo postoperative (day 80 after second lenticule extraction)	1.2/1.2	Clear cornea	Normal	Mild myopia in the right eye (−0.25/ −0.25 × 170); mild myopia with slight astigmatism in the left eye (+0.00/ −0.50 × 10)	No additional treatment needed	

## 3. Discussion

Diffuse lamellar keratitis is a significant postoperative complication associated with refractive surgeries, particularly laser-assisted procedures such as SMILE.^[7]^ Characterized by an inflammatory response in the corneal stroma, DLK can severely compromise visual outcomes and patient satisfaction if not addressed promptly. The etiology of DLK remains complex, with various factors contributing to its onset, including retained lenticules, which may act as foreign bodies, inciting an inflammatory cascade that leads to corneal opacity and discomfort.^[8]^ Understanding the pathophysiology and risk factors associated with DLK is crucial for optimizing surgical techniques and postoperative care.

This study investigates the correlation between delayed-onset DLK and retained SMILE lenticules through a detailed case report and a comprehensive literature review. The case illustrates a patient who developed DLK symptoms postoperatively, highlighting the clinical manifestations, treatment protocols, and ultimate recovery outcomes.^[9,10]^ Our findings underscore the necessity for vigilant monitoring and prompt intervention in cases of retained lenticules to mitigate the inflammatory response and facilitate visual recovery. Future research should delve deeper into the underlying mechanisms of DLK and establish evidence-based guidelines for managing this complication effectively, thereby improving patient outcomes post-SMILE surgery.

The findings of this study provide significant insights into the association between delayed-onset DLK and retained lenticules following SMILE surgery. Unlike previous research, which primarily focused on the incidence and management of epithelial-related complications after SMILE, this study uniquely emphasizes the role of retained lenticules as potential foreign bodies inducing inflammatory responses in the cornea, particularly evident in our case report where DLK developed on postoperative day 5. Previous studies have documented cases of postoperative complications but lacked a focused exploration of the correlation between retained lenticules and DLK, thereby leaving a critical gap in the literature. Our findings corroborate the notion posited by Wilson et al (2020), which suggests that retained tissues can indeed provoke inflammatory reactions, but our case provides the first human evidence linking DLK specifically to retained SMILE lenticules, paving the way for further exploration into this phenomenon.^[11]^

The implications of our findings extend to clinical practice and policy formulation regarding postoperative management after SMILE procedures. The prompt identification and treatment of DLK, as demonstrated by the successful intervention in our case, underscore the necessity for clinicians to maintain a high index of suspicion for this condition in the immediate postoperative period. This aligns with the recommendations of recent reviews that advocate for vigilant monitoring of patients for potential complications associated with retained lenticules. Moreover, establishing a standardized protocol for the management of DLK may enhance patient outcomes and satisfaction, particularly in a landscape where refractive surgeries are becoming increasingly common. As such, our study not only contributes to the understanding of DLK but also highlights the need for improved clinical guidelines that can directly influence patient care post-SMILE surgery.

However, this study is not without its limitations. The case report format naturally restricts the generalizability of findings, as it is based on a single patient’s experience. Additionally, the literature review, while comprehensive, may not encompass all potential studies and reports on the topic, thus potentially overlooking relevant data that could further illuminate the relationship between retained lenticules and DLK. Future research should aim to include larger sample sizes and multi-center studies to validate our findings and assess the long-term outcomes of patients with retained lenticules post-SMILE surgery. By addressing these limitations, researchers can enhance the understanding of DLK’s pathogenesis and develop more effective management strategies, ultimately improving patient safety and visual outcomes in refractive surgery practices.

This study is subject to several limitations that must be acknowledged. The reliance on a single case report, while providing detailed insight into the clinical manifestations and management of DLK following SMILE surgery, restricts the generalizability of the findings. The unique characteristics of the reported patient may not represent the broader population of individuals undergoing SMILE procedures. Additionally, the literature review, although comprehensive, may be susceptible to publication bias, as studies with negative or inconclusive results are less likely to be published. Furthermore, the retrospective nature of the review limits the ability to draw causal inferences regarding the relationship between retained lenticules and DLK. Future studies with larger sample sizes and prospective designs are warranted to validate these findings and elucidate the underlying mechanisms that contribute to DLK development in post-SMILE patients.

## 4. Conclusion

The evidence presented in this study underscores the critical association between delayed-onset DLK and retained SMILE lenticules, emphasizing the need for heightened awareness among ophthalmic practitioners. Prompt identification and management of DLK are paramount to fostering optimal visual recovery and enhancing patient satisfaction following refractive surgery. This investigation highlights the necessity for further research to delineate the pathophysiological mechanisms and risk factors associated with DLK, thereby informing improved postoperative strategies. A deeper understanding of these aspects will ultimately contribute to enhanced clinical outcomes and the development of tailored treatment protocols for patients experiencing this complication post-SMILE surgery.

## Acknowledgments

We would like to express our sincere gratitude to all individuals who contributed to the research and manuscript preparation. Special thanks to GF and HY for their invaluable assistance and support throughout the study. We also appreciate the significant contributions of WY and ZW to the development of the research. Any individuals who contributed to the research or manuscript but are not listed as authors have been acknowledged with their permission.

## Author contributions

**Data curation:** Hai Yu, Wei Yang.

**Formal analysis:** Jiaxing Pan.

**Methodology:** Guangbi Fu, Jiaxing Pan, Hai Yu, Wei Yang.

**Software:** Guangbi Fu.

**Supervision:** Zhengzheng Wu.

**Writing – original draft:** Guangbi Fu.

**Writing – review & editing:** Zhengzheng Wu.
